# Scalable production of pectinases from *Bacillus licheniformis* SMIA-2 using agro-Industrial by-products with genomic insights

**DOI:** 10.1007/s10068-026-02252-3

**Published:** 2026-07-25

**Authors:** Erica Cruz, Larissa Pacheco Ferreira, Albert Remus Romero Rosana, Samara Pinto Custódio Bernardo, Meire Lelis Leal Martins, João Paulo Fabi

**Affiliations:** 1 Department of Food Science and Experimental Nutrition, School of Pharmaceutical Sciences, University of São Paulo, São Paulo, Brazil; 2 Food Technology Laboratory, State University of North Fluminense, Campos dos Goytacazes, Rio de Janeiro, Brazil; 3 Department of Biology, De La Salle University, Taft Avenue, Manila, The Philippines; 4 Food Technology Laboratory, State University of North Fluminense, Campos dos Goytacazes, Rio de Janeiro, Brazil; 5 Food and Nutrition Research Center (NAPAN), University of São Paulo, São Paulo, Brazil; 6 Food Research Center (FoRC), CEPID-FAPESP (Research, Innovation and Dissemination Centers, São Paulo Research Foundation), São Paulo, São Paulo Brazil

**Keywords:** Pectinases, *Bacillus licheniformis*, Enzyme production, Agro-industrial residues, Spray drying, Enzyme stabilization

## Abstract

The study re-analyzed the draft genome of *Bacillus licheniformis* SMIA-2 and generated a reference-guided pseudo-scaffold. Cross-validated genome annotation identified five candidate loci associated with pectin degradation, including putative pectate lyases, polygalacturonase, and downstream uronate-catabolic genes. Submerged fermentation with passion fruit peel flour and corn steep liquor yielded crude enzymatic extracts, which were spray-dried at 110 °C using maltodextrin and microcrystalline cellulose as stabilizers. The dried formulation retained pectinase activity for 180 days at 5 °C and showed additional cellulase, amylase, xylanase, and protease activities. Pectinase displayed optimal activity at pH 8.5 and 70 °C, with stability between pH 8.0–8.5 and 65–70 °C. Despite not using a reference strain and the absence of some omics analyses, with genomic and industrial claims presented as evidence of biotechnological potential rather than definitive functional validation of individual genes, these results support a sustainable, scalable, and alkaline-tolerant enzyme platform based on agro-industrial residues.

**Supplementary Information:**

The online version contains supplementary material available at https://doi.org/10.1007/s10068-026-02252-3.

## Introduction

Generally recognized as safe (GRAS), *Bacillus licheniformis* is a Gram-positive, spore-forming bacterium that can be easily isolated from soil and plant samples, making it of considerable biotechnological relevance. This species is known for synthesizing a range of bioactive compounds with applications across diverse industrial sectors, including aquaculture, agriculture, food technology, biomedicine, and the pharmaceutical industry (Medison et al., 2023). The industrial interest in *Bacillus*-derived enzymes is partly due to their capacity for secretion, relatively short fermentation times, and frequent activity under neutral, alkaline, or elevated-temperature conditions. These characteristics are useful for biomass processing, detergent formulations, textile applications, pulp and paper processing, and food bioprocessing, where enzyme robustness can reduce process costs and simplify downstream operations (Muras et al., 2021; Zhou et al., 2024).

Pectinases comprise enzymes that modify or depolymerize pectic polysaccharides by hydrolytic or lyase mechanisms. Major pectin-degrading activities include polygalacturonases, pectate lyases, pectin lyases, pectin esterases, rhamnogalacturonan lyases, galactanases, and arabinofuranosidases (Patidar et al., 2018). Pectinase preparations are widely used for fruit juice clarification, vegetable maceration, coffee and tea processing, plant-fiber treatment, and pulp processing (Koziol et al., 2017).

*Bacillus licheniformis* SMIA-2 is a strictly aerobic, thermophilic, spore-forming bacterium isolated from soil samples collected in Campos dos Goytacazes, Rio de Janeiro, Brazil (Souza and Martins 2001). Previous work reported that SMIA-2 can produce hydrolytic enzymes, including proteases, xylanases, pectinases, avicelases, and carboxymethylcellulases (Bernardo et al., 2020). However, the relationship between this phenotype and the genome-encoded repertoire for pectin degradation had not been analyzed in detail.

Passion fruit peel flour was selected as a carbon-rich substrate because it is abundant, low-cost, and rich in pectin and other plant cell-wall polymers. Corn steep liquor was used as a low-cost nitrogen source that provides peptides, amino acids, vitamins, phosphate, and metal ions. The use of these residues is consistent with circular bioprocessing strategies that valorize underutilized agro-industrial streams while potentially reducing enzyme production costs (Cano-González et al., 2024; Martins et al., 2020; Sadh et al., 2018).

Drying crude enzyme preparations can improve storage stability, handling, and transport. Spray drying is economically attractive compared with freeze-drying because it processes larger volumes with higher throughput, but protein denaturation during atomization and exposure to hot air must be minimized (Jesus and Maciel Filho, 2014; Schutyser et al., 2012). Carbohydrate and polymeric carriers, including maltodextrin and microcrystalline cellulose, can increase solids content, reduce caking, and protect heat-sensitive compounds during drying (Assadpour and Jafari, 2019; Ohtake et al., 2011).

The objectives of this study were to re-analyze the publicly available draft genome of SMIA-2 to identify candidate pectinase-related genes and pathways; to evaluate pectinase activity in a crude enzyme preparation produced with passion fruit peel flour and corn steep liquor; and to assess the feasibility of stabilizing this crude preparation by spray drying. The study emphasizes process feasibility and genomic potential, while explicitly recognizing that direct gene expression, secretion, purified enzyme, and commercial benchmark experiments remain necessary for full functional validation.

## Materials and methods

### Bioinformatic analysis of *Bacillus licheniformis* SMIA-2 genome

Genomic analyses were based on in silico annotation and bioinformatic prediction approaches. The genome of *Bacillus licheniformis* SMIA-2 (referred to as SMIA-2 from here on) was previously sequenced at the Molecular Biology Service Unit of the Department of Biological Sciences, University of Alberta, Canada, using the Illumina paired-end sequencing platform (Illumina, USA). The whole-genome sequence was retrieved from NCBI GenBank under accession number JAACZZ000000000 (Bernardo et al., 2020). The focus of this genome analysis is to probe the putative pectinases or degradation enzymes (pectate lyases, pectin esterases, hydrolases, polygalacturonase, etc.) encoded in the draft genome to corroborate the phenotypic characteristics observed in SMIA-2, which exhibit high pectinase activity.

To increase the predictive power of genome analysis, we utilized the draft genome 34 contiguous sequences to perform independent annotations and cross-validated gene calling pipelines, namely: (1) the NCBI-PGAP, (2) JGI-IMG/M, (3) RASTtk, and (4) ProkSee (Daas et al., 2018). For visualization purposes, the contigs were re-aligned with respect to the closed genome and type strain, *Bacillus licheniformis* Gibson 46^ T^ (ATCC 14580), using Contiguator ver2 and MEDUSA. The chromosome map was generated from the resulting single scaffold using CGViewer (Grant et al., 2023). Default parameters were used for all software unless otherwise specified. A manual secondary search was performed using the EC (Enzyme Commission) numbers of protein sequences associated with pectin metabolism. The protein sequences were searched using tBLASTn with *B. licheniformis* Gibson 46 T as the query genome and cross-validated in the *B. licheniformis* SMIA-2 proteome.

Gene loci with direct annotation calls for pectate lyase, pectin esterase, pectinase, or poly-D-galacturonase were retrieved, and each gene product was further validated using BLASTp, and the protein structure was cross-referenced with the closest crystal structure using the ExPASy Swiss-Model server. The gene loci were re-labeled as the pectinase gene in Proksee's CGViewer map, along with adjacent genes, to provide potential insights into gene/operon conservation and/or metabolic or evolutionary synteny.

The metabolic gene inventory of SMIA-2 associated with pectin degradation was further analyzed using the Seed Viewer package as implemented in the RAST server. The general subsystem features of the SMIA-2 genome (e.g., carbohydrates, polysaccharides, oligosaccharides) were generated, and a comparative pectinase/poly-D-galacturonase operon analysis was performed with particular focus on thermophilic/thermoduric bacterial species showing relevant pectin degradation genes. The comparison of SMIA-2 predicted proteome composition was performed with closely related *Bacillus* species (Daas et al., 2018), including *B. licheniformis* Gibson 46^ T^, with *Lactiplantibacillus plantarum* LB1-2 as the outgroup (Ilagan-Cruzada et al., 2018). Comparison of the proteome maps of SMIA-2 and four closely related *Bacillus* species by uni- and bidirectional best BLASTp searches implemented in RAST, cross-validated against IMG annotations, was performed.

To further support the genomic blueprint and proteomic capacity for a putative pectin degradation module in SMIA-2, a metabolic reconstruction of the pectin/poly-D-galacturonate pathway was probed using the KEGG Metabolic Comparison Tool. This includes the following modules: (1) pectin degradation (M00081), (2) D-galacturonate degradation (bacteria) (M00631), and (3) D-glucuronate degradation (M00061). This study did not elaborate further on the interlinkages of the SMIA-2 pectin degradation pathway towards the metabolism of amino and nucleoside sugars. Moreover, the orthologous fungal pectin degradation (M00630) pathways were also excluded.

The protein structures of the candidate pectinases were further analyzed using AlphaFold DB, and ExPASy Swiss-Model server, with default parameters. To provide a robust inference on the probable secreted pectinases during SMIA-2 fermentation, the signaling potential of the SMIA-2 pectinases was probed using SignalP 6.0 while the protein subcellular localization was predicted using DeepProLoc ver1.0. Other biochemically characterized enzymes associated with pectin catabolism were used as subcellular localization controls.

### Microorganism and culture conditions

The bacterial strain used in this study was *Bacillus licheniformis* SMIA-2, isolated from a soil sample collected in Campos dos Goytacazes, Rio de Janeiro, Brazil (Souza and Martins, 2001). The production medium contained (g L⁻^1^): KCl, 0.30; MgSO₄, 0.50; K_2_HPO₄, 0.87; CaCl_2_, 0.29; ZnO, 2.03 × 10⁻^3^; FeCl_3_·6H_2_O, 2.70 × 10⁻^2^; MnCl_2_·4H_2_O, 1.00 × 10⁻^2^; CuCl_2_·2H_2_O, 8.50 × 10⁻^4^; CoCl_2_·6H_2_O, 2.40 × 10⁻^3^; NiCl_2_·6H_2_O, 2.50 × 10⁻^4^; H_3_BO_3_, 3.00 × 10⁻^4^; commercial corn steep liquor, 3.0; and passion fruit peel flour, 6.0. The pH was adjusted to 7.5 with 1.0 M NaOH, and the medium was sterilized at 121 °C for 15 min.

Fifty milliliters of medium in 250 mL Erlenmeyer flasks was inoculated with 1 mL of a standard overnight culture to obtain an initial cell concentration of approximately 10^4^ CFU mL⁻^1^. Cultures were incubated for 72 h at 50 °C and 150 rpm. After incubation, the fermentation broth was passed through a Granutest sieve (Tyler 250 mesh; 63 µm pore size) to remove large particles, residual substrate, and bacterial aggregates. This sieving step was not designed to remove individual bacterial cells because the mesh pore size exceeds the typical size of Bacillus cells. The filtrate was therefore defined as a crude enzyme preparation containing extracellular enzymes, soluble fermentation components, and possible suspended bacterial cells or spores.

Centrifugation and membrane filtration were not included in the process workflow to maintain a simple, low-cost downstream operation and to reduce potential activity losses associated with additional handling. This choice is compatible with an industrial screening strategy for crude enzyme preparations, but it also means that residual viable cells, spores, or cell-associated enzymes could have contributed to measured activity or apparent storage stability. This limitation was considered in the interpretation of the results.

### Enzyme assay

Pectinase activity was determined with the dinitrosalicylic acid (DNS) reducing-sugar assay, using D-galacturonic acid as the standard (Supplementary Fig. 1A; Miller, 1959). Results are expressed as galacturonic-acid-equivalent reducing activity under the stated assay conditions rather than as an absolute molar turnover value for a purified enzyme. The pectinase reaction mixture contained 0.5 mL of 1% (w/v) apple pectin prepared in 0.05 M glycine–NaOH buffer (pH 8.5) and 0.5 mL of appropriately diluted crude enzyme preparation. Reactions were incubated at 70 °C for 10 min, stopped with 1.0 mL DNS reagent, heated for 5 min, cooled to room temperature, and read at 540 nm. One unit (U) of pectinase activity was defined as the amount of enzyme releasing 1 µmol of galacturonic-acid-equivalent reducing groups per minute under the assay conditions. When the activity was tested using Avicel as the substrate, one unit (U) of activity toward the substrate was defined as the amount of glucose-equivalent released per minute under the above assay conditions, determined from a glucose standard curve (Supplementary Fig. 1B). Enzyme blanks, substrate blanks, and buffer controls were run in parallel and subtracted from sample absorbance values. The pectinase assay contained pectin as the only added polysaccharide substrate; cellulose, starch, and xylan were not present in this reaction. Therefore, reducing sugars produced from those substrates could not directly contribute to the pectinase assay signal. However, the crude enzyme preparation contained multiple hydrolytic activities, and the DNS method can respond differently to different reducing products. The results should therefore be interpreted as pectin-directed reducing activity in a crude preparation rather than as the kinetics of purified pectinase. Additional activities toward Avicel, carboxymethylcellulose, starch, xylan, and protein substrates were measured separately to characterize the broader hydrolytic profile of the crude preparation.

### Spray drying of crude enzyme solution

Spray-drying parameters were selected from preliminary optimization experiments performed with SMIA-2 enzyme preparations and from the literature describing spray-drying of enzyme-containing formulations (Cruz, 2021; Libardi et al., 2020; Schutyser et al., 2012). The selected conditions balanced powder recovery, process simplicity, and retention of pectinase activity while using low-cost, food-compatible carrier agents. The crude enzyme preparation was mixed with 0.5% (w/v) maltodextrin and 1.0% (w/v) microcrystalline cellulose and dried in a laboratory-scale spray dryer (LAB-PLANT SD-04, England). A 100 mL feed volume was supplied at 2.5 mL min⁻^1^ through a peristaltic pump. Drying air flowed co-currently with the atomized droplets. The inlet temperature was 110 °C, and the outlet temperature was 69 °C. The airflow rate was kept constant. Drying experiments were performed in duplicate. Pectinase activity was measured before and after drying. Dried powder was reconstituted to 0.01 g mL⁻^1^ prior to enzymatic analysis, and residual activity was calculated relative to the crude enzyme preparation prior to drying. Dried samples were stored at 5 °C for up to 180 days to assess stability. The study evaluated residual activity but did not include SDS-PAGE, zymography, mass spectrometry, or other direct measurements of protein integrity before and after spray drying.

### Statistical analysis

Data are presented as mean ± standard deviation from triplicate assays unless otherwise specified. Statistical analyses were performed using SAS software and Microsoft Excel. One-way analysis of variance (ANOVA) was used to compare experimental conditions, followed by Tukey’s post hoc test when multiple comparisons were required. Normality and homogeneity of variance were assessed prior to ANOVA. Differences were considered significant at *p* < 0.05.

## Results and discussion

### Genomic potential for pectin degradation in a draft genome context

**Fig. 1:**
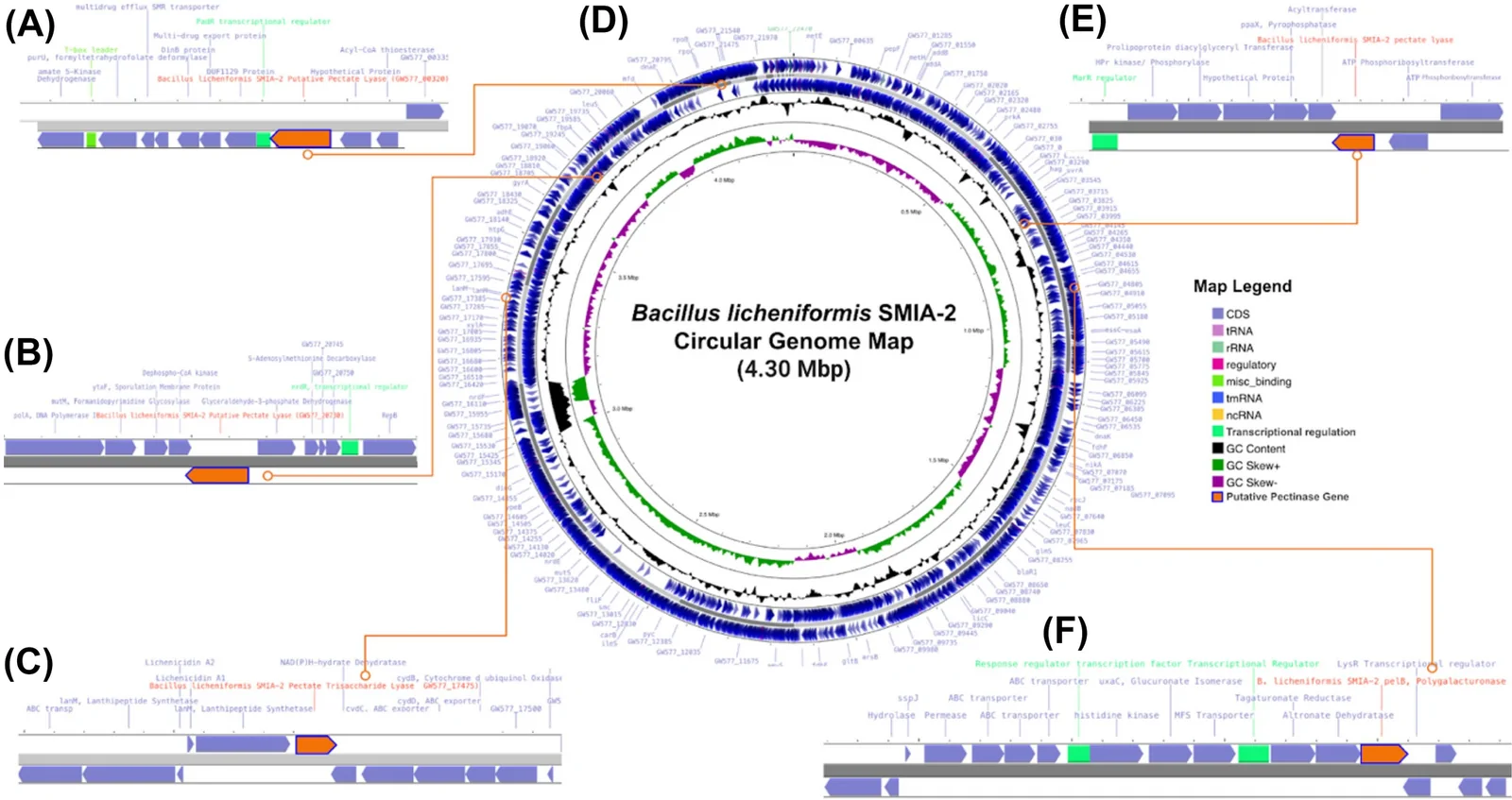
Graphical representation of the whole genome map of *Bacillus licheniformis* SMIA-2 and the five putative pectin catabolic gene loci. Putative pectinase gene clusters were mapped from the assembled draft genome using ProkSee visualization tools: (**A**) putative extracellular *pel*A-like pectate lyase organized with the glutamate metabolism gene clusters, (**B**) a homolog of *pelB*-like pectate lyase, (**C**) a putative tri-saccharide pectate lyase gene clustered with the two-component secondary metabolite lantibiotic, lichenicidin A1/A2, biosynthetic gene cluster, the circular map (**D**) of the draft genome of SMIA-2 represents relevant genome features, (**E**) a *rhiN*-like putative pectate lyase gene, and (**F**) a endo-poly-D-galacturonase gene homolog organized in an operon with several galactorunate metabolism associated genes. The draft genome was aligned to a single scaffold using the type strain. *B. licheniformis* Gibson46^T^. The lines in each concentric circle represent the position of the indicated feature; the color legend is shown to the right of the map. The first circle indicates the predicted coding regions transcribed on the forward (clockwise) DNA strand. The second circle shows the predicted coding regions transcribed on the reverse (counterclockwise) strand of DNA. The third and fourth circles show the genome-wide %GC content and strand-specific % GC skewness, respectively

Five genomic loci were identified with gene annotations of pectate lyase or poly-D-galacturonase gene (Fig. 1, Table 1). Among the five putative pectate genes, *pelA* and *pelB* showed high sequence homology with known pectinase genes and were found to be part of their respective gene clusters. The SMIA-2 *pelA*-like gene homolog is organized in a gene cluster involved in glutamate metabolism (Fig. 1A). Conversely, the *pelB*-like gene homolog is clustered with the biosynthetic gene cluster for lichenicidin A1/A2, a two-component lantibiotic peptide (Fig. 1C). The *pelB*-like gene homolog in SMIA-2 encodes a trisaccharide pectate lyase, and the closest hit is an exo-polygalacturonate lyase (Table 1). The two other pectate lyase genes are found in separate genomic regions, separate from the cluster they are associated with (Fig. 1B, E). The gene locus GW577_03585 encodes a putative pectate lyase C-like enzyme or a polygalacturonic transeliminase (EC 4.2.2.2), while GW577_20730 encodes a putative poly-methyl-galacturonic transeliminase (EC 4.2.2.10). A putative endo-polygalacturonase gene (GW577_04685) was retrieved from the genome, encoding a protein with sequence homology to glycoside hydrolase family 28 (EC 3.2.1.67), and is arranged in a 13-gene operon. The gene cluster-encoded enzymes are involved in key intermediary pathways downstream of pectin degradation, including altronate dehydratase, tagaturonate reductase, and glucuronate isomerase (Fig. 1F, Table 1). Previous studies have reported similar arrangements in *Bacillus* and *Paenibacillus* strains, reinforcing the biotechnological relevance of this microbial group (Ouattara et al., 2011).

**Table 1:** Putative pectate lyase genes retrieved from the draft genome of *Bacillus licheniformis* SMIA-2

*Bacillus licheniformis* SMIA-2 Putative Pectinase Genes			NCBI closest protein hit	Closest reference bacterial hit	GenBank/ NCBI Ref sequence	Sequence homology (%)	e-Value
Gene	Gene locus	Protein locus					
pelA-like	GW577_00320	NCL89449.1	Pectate lyase family protein	*B. licheniformis* Gibson 46^ T^	AAU22955.1	100	0.00
Putative pectate gene	GW577_20730	NCL93387.1	RICIN-domain Pectate lyase	*Bacillus* sp. PAMC28748	QNH43934.1	100	0.00
Pectate trisaccharide lyase (pelB-like)	GW577_17475	NCL92780.1	Exo-poly- galacturonate lyase	*B. licheniformis* Gibson 46^ T^	Q65DC2.1	100	0.00
Putative pectate gene (rhiN-like)	GW577_03585	NCL90084.1	Pectate lyase Pel-28 K	*B. licheniformis* WX-02	AKQ75006.1	100	0.00
Endo polygalacturonase	GW577_04685	NCL90296.1	Glycoside hydrolase family 28	*B. licheniformis* WX-02	AKQ74773.1	100	0.00

### Subsystem distribution and operon synteny of pectin-degradation genes in SMIA-2

**Fig. 2:**
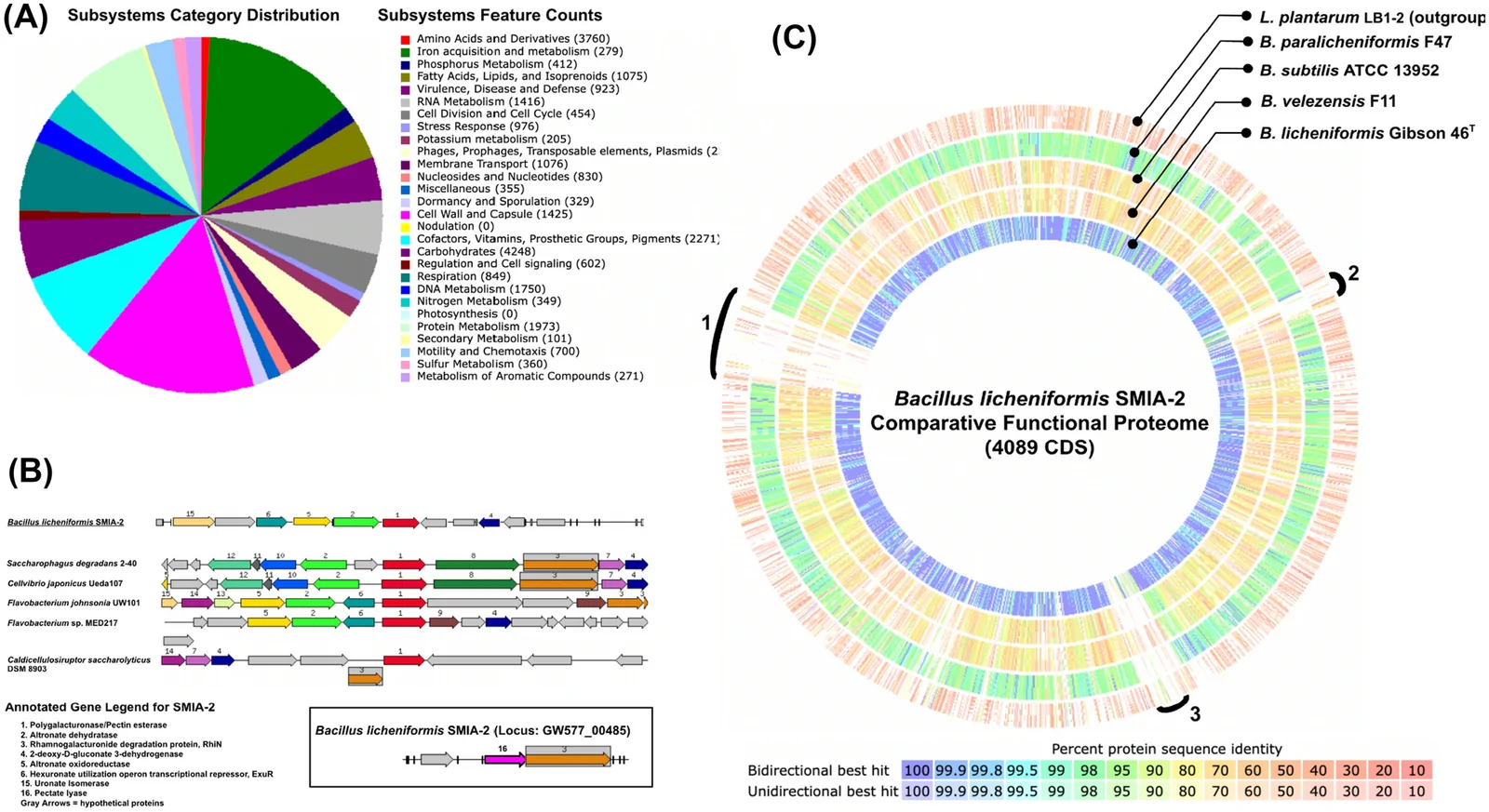
Genome analysis of *Bacillus licheniformis* SMIA-2 using the RAST annotation pipeline. (**A**) Genes’ association with functional subsystems and their distribution in different metabolic categories. The categories are expandable down to the specific gene through feature counts, including the carbohydrate subsystem category “D-galacturonate and D-glucuronate utilization”. (**B**) The annotated and predicted intracellular poly-D-galacturonase/pectin esterase gene aligned through comparative gene cluster analysis with other thermophilic bacterial species and gene synteny with the galacturonate catabolism-associated gene operon (numbered and color-coded for the homologs). The predicted extracellularly targeted pectin lyase, *pelA*-like gene, is outside the operon (inserted box). (**C**) A comparative proteome circular map of *B. licheniformis* SMIA-2 with other *Bacillus* species was conducted using the RAST pipeline. Each track represents a pair-wise BLASTp comparison between closely related species against those of the 4,089 coding sequences (CDS) of SMIA-2, with the percentage of similarity represented with different colors shown in the legend. Query genomes used in this analysis (inner ring to outer ring): *B. licheniformis* Gibson 46^ T^, *B. velezensis* F11, *B. subtilis* ATCC 13952, *B. paralicheniformis* F47, and *Lactiplantibacillus plantarum* LB1-2 (outgroup). Regions labelled as 1, 2, and 3 correspond to the three major genomics islands associated with phage gene cluster insertion sites

*Bacillus licheniformis* SMIA-2 showed not just high pectinase activity but also other enzymatic activities involving complex polysaccharide/oligomeric substrate degradation (Bernardo et al., 2020) (Table 1). The RAST-annotated genome revealed a large gene set for carbohydrate and polymeric substrate metabolism, with approximately 4248 associated gene features (Fig. 2). This extensive list revealed gene features for the metabolism of fructooligosaccharides and genes encoding enzymes such as amylase, cellulase (avicelase), and xylanase (Fig. 2A). The sub-feature category directly linked to pectin degradation includes the monomeric subunits D-galacturonate and D-glucuronate, with about 124 gene features. Only one gene was identified as associated with the D-galacturonate utilization module: the endo-polygalacturonase gene GW577_04685.

The subsystem category distribution (Fig. 2A) showed a predominance of genes associated with carbohydrate metabolism. This result suggests the microorganism’s adaptive capacity to degrade complex substrates, consistent with its environmental origin and thermophilic profile. The endo-polygalacturonase gene of SMIA-2 revealed an interesting operon synteny with several pectin– (and polysaccharide-degrading bacteria (Fig. 2B), albeit not immediately with the closest *Bacillus* species (Fig. 2C). The observed synteny includes gene clusters from the strains *Saccharophagus degradans* 2–40, *Cellvibrio japonicus* Ueda107, *Flavobacterium johnsonia* UW101, *Flavobacterium* sp. MED217, and *Caldicellulosiruptor saccharolyticus* DSM 8903. The other functional genes revealed homology to altronate dehydratase, 2-deoxy-D-gluconate 3-dehydrogenase, altronate oxidoreductase, hexuronate utilization operon transcriptional repressor, and uronate isomerase. Interestingly, one of the key genes involved in downstream pectin catabolism, *rhiN*, which encodes rhamnogalacturonidase, is not in the same operon in SMIA-2 but is an independent gene located far from the operon scaffold and is still annotated as a pectate lyase (Fig. 2B, box insert). Only the closest type strain, *B. licheniformis,* demonstrated a high proteome sequence similarity with a uni/bi-directional protein hit of > 99.8%, followed by *B. paralicheniformis* (95–98%), *B. subtilis—B. velezensis* (60–80%), and *Lactiplantibacillus plantarum* (10–50%). Although *B. licheniformis* SMIA-2 is evolutionarily clustered as a member of the *B. subtilis-amyloliquefaciens-licheniformis *sensu lato, the pectate lyase genes are not organized into a syntenic operon (Fig. 2C, regions 1, 2, and 3), which may represent possible sites of recombination/speciation. This could be the case for the non-operonic putative gene *rhiN*, which codes for rhamno-galacturonidase (GW577_03585), as it is located in genomic island region 2 (Fig. 2C) rather than within the canonical pectate lyase operon (Fig. 2B).

### kegg-based reconstruction of pectin catabolism and secretion features of SMIA-2 pectate lyases

**Fig. 3:**
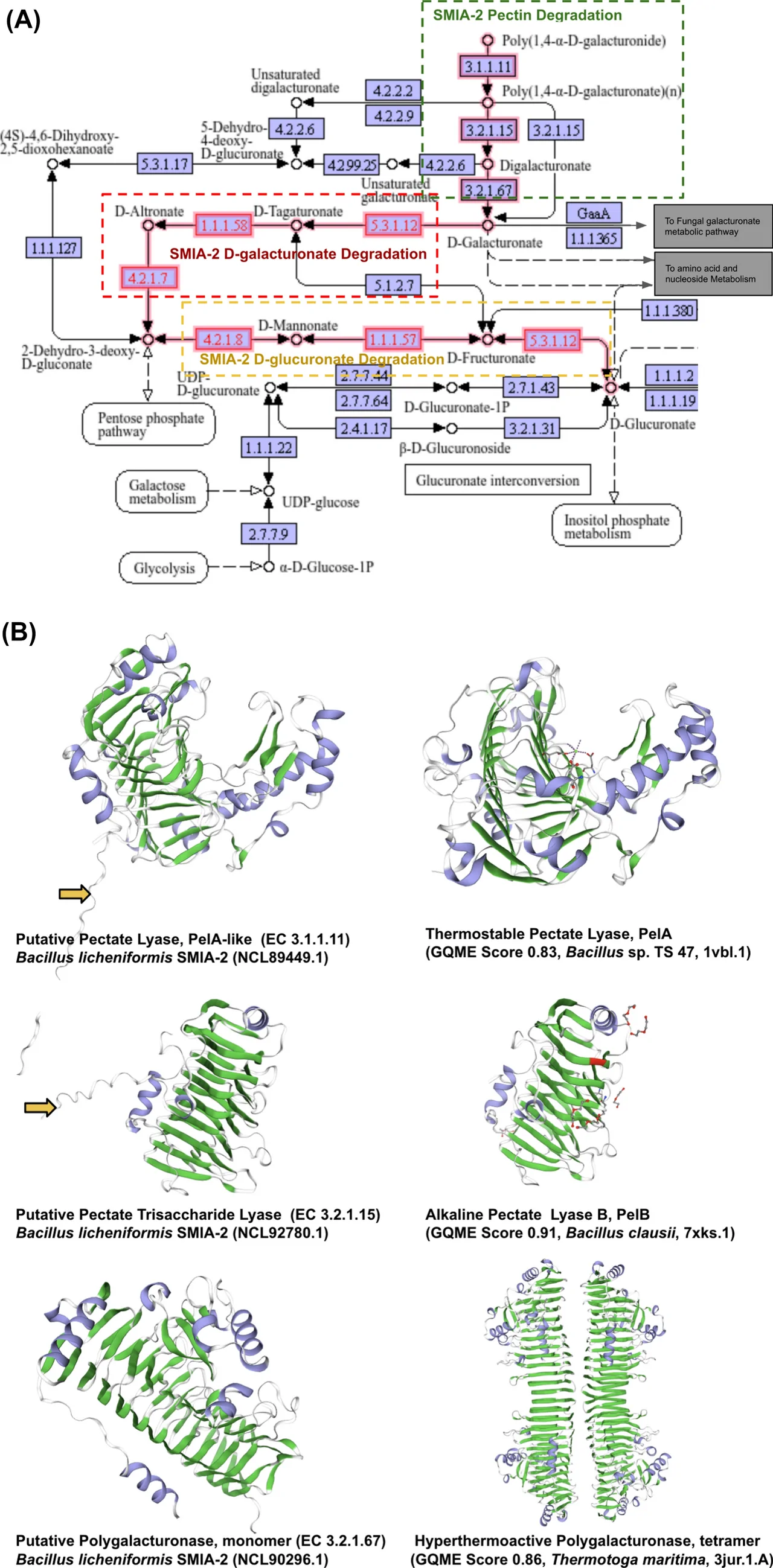
Metabolic reconstruction of the functional pectin degradation pathway encoded in the genome of *B. licheniformis* SMIA-2 modeled using the KEGG pathway analysis module. (**A**) The relevant enzymes (EC numbers) involved in the pathways supported by the encoded annotated genes (highlighted in pink boxes). The pectin metabolic pathways were divided into three modules: pectin/poly-D-galacturonate degradation module highlighted in green dotted box, D-galacturonate catabolism module in red dotted box, and D-glucuronate metabolism in yellow dotted box. (**B**) The structure of the three putative key enzymes in SMIA-2 pectin degradation was modeled using AlphaFoldDB and the corresponding X-ray crystal structure of the experimentally verified and bonafide pectinases. The putative pectinases were predicted to be extracellularly secreted (i.e., PelA-like and pectate trisaccharide lyase) with the signaling N-terminal protein domain indicated by the yellow arrow. The crystal structure of the corresponding enzyme template was shown together with the active site or substrate, either in monomeric or tetrameric form. The corresponding protein EC numbers were provided, along with the GQME scores, the reference bacterial species, and the SMTL–D codes. The SMIA-2 pectate lyases amino acid Genbank numbers are in parentheses

The KEGG-based metabolic reconstruction of the SMIA-2 revealed a complete pathway from the degradation of the pectin to its monomeric unit, D-galacturonate. The enzymes involved in downstream pathways linked to other sugar catabolic pathways (i.e., the pentose phosphate and glycolytic pathways) were also represented in the genome (Fig. 3A). At least three of the five pectate lyase genes encoded in the genome (GW577_00320, GW577_17475, and GW577_04685) support the initial steps of the pectin degradation module (M00081), leading to the monomeric D-galacturonate. The bacteria-specific D-galacturonate degradation module (M00631) shows complete gene loci leading to 2-dehydro-3-deoxy-D-gluconate, all of which were detected as an operon in SMIA-2 (Fig. 2B): β-galacturonate isomerase, D-tagaturonate reductase, and β-altronate dehydratase. Lastly, the subsequent third module (M00061) allowed for the conversion of 2-dehydro-3-deoxy-D-gluconate to D-glucuronate in three inversion reactions governed by SMIA-2’s genome-encoded mannonate dehydratase, fructuronate reductase, and glucuronate isomerase (Fig. 3A).

Analysis of the signaling and subcellular localization of the translated proteins from all five pectate lyase genes revealed that all enzymes, except the endo-poly-D-galacturonase, were potentially subject to post-translational control via a putative Sec-dependent secretion system. This supports the possibility of secreted exo-enzymes via a putative, conserved Sec-dependent peptidase cleavage site (Ala-X-Ala motif). The subcellular localization analysis yielded a confidence score of at least 0.99 for an extracellular secretion profile for all four SMIA-2 pectate lyases. The N-terminal signaling domain–analyzed with AlphaFold DB-indicates that the unfolded N-terminus is a putative signal peptide (Fig. 3B, yellow arrows).

### Pectinase production in agro-industrial medium and interpretation of crude-extract activities

Although the primary focus of the study was the production and characterization of pectinase activity by *Bacillus licheniformis* SMIA-2, additional enzymatic activities were evaluated, as the crude enzymatic extract from agro-industrial substrate fermentation may naturally contain a consortium of carbohydrate-degrading enzymes. The crude enzymatic extract exhibited enzymatic activity toward other substrates, as shown in Table 2. In addition to pectinases, the crude enzymatic extracts showed activities of avicelase (0.049 U/mL), carboxymethylcellulase (0.041 U/mL), amylase (0.137 U/mL), xylanase (0.022 U/mL), and protease (6.300 U/mL). According to Van et al. (2009), different enzymes are necessary for the degradation of complex substrates present in the plant cell wall. Some organisms express free enzymes, while others produce large extracellular multienzyme complexes.

**Table 2:** Enzymatic activity of the crude extract obtained after 72 h of fermentation is present in the crude extract of *Bacillus licheniformis* SMIA-2

Enzyme	Enzymatic activity (U/mL)
Pectinase	18.431^a^ ± 0.002
Avicelase	0.049^d^ ± 0.001
Carboxymethylcellulase	0.041^e^ ± 0.001
Amylase	0.137^c^ ± 0.003
Xylanase	0.022^f^ ± 0.002
Protease	6.300^b^ ± 0.002

^1^Values are expressed as mean ± standard deviation. Means followed by different superscript letters differ significantly according to Tukey’s test (p ≤ 0.05)

Passion fruit peel flour, in addition to containing cellulose in its composition, is rich in pectin (21.5%), tryptophan, fatty acids, and amino acids (Guertzenstein and Srur, 2002). Corn steeping water contains amino acids, peptides, vitamins, phosphate, and metal ions (Rivas et al., 2004). Therefore, considering the composition of the culture medium used to grow *Bacillus licheniformis* SMIA-2, which is used for the production of pectinases, the importance of the secretion of the multienzyme complex for the degradation of all nutrients present in the medium can be highlighted. By using an enriched culture medium containing a pectin-rich substrate, such as passion fruit peel flour, we stimulated bacterial production of pectinases. This stimulation may be due to the bacteria’s metabolic needs, since other essential substrate concentrations were lower than in the usual protocols, thereby triggering energy-dependent metabolic pathways, or simply to the bacteria’s contact with pectins, which triggered the production of pectinases. Further studies are needed to identify the specific biochemical events.

### Spray-dried formulation and stability

Given the industrial relevance of thermostable pectinases, we sought to extend the shelf life of pectinase solutions produced by *Bacillus licheniformis* SMIA-2 using spray drying. Pectinases were recovered from submerged cultures of SMIA-2 containing passion fruit peel flour and corn steep liquor after 72 h. Pectinase activity (pectin-hydrolyzing enzymes) was prominent in the supernatant compared to the other enzymes (avicelase, carboxymethylcellulase, amylase, xylanase, and protease). Table 3 shows the activity of the enzymes present in the dry (crude enzymatic extract, ES) obtained from the supernatant (crude pectinase extract, CPS). The ES was obtained by spray drying the crude enzymatic extract from cultures of *Bacillus licheniformis* SMIA-2, which contained passion fruit peel flour and corn steeping water. The stability of the pectinase powder obtained during storage at 5 °C for up to six months was investigated (Supplementary Table 1). Table 3 shows the effect of storage time on pectinase activity after spray drying in the presence of 0.5% (w/v) MD and 1.0% CM, at an atomizer inlet temperature of 110 °C. Enzymatic activity was completely preserved when stored at 5 °C.

**Table 3:** Enzymatic activity of the dry crude extract (ES) obtained after spray drying by the spray dryer

Enzyme	Enzymatic activity (U/mL)
Pectinases	13.656^a^ ± 0.003
Avicelase	0.043^c^ ± 0.001
Carboxymethylcellulase	0.038^d^ ± 0.001
Amylase	0.023^e^ ± 0.003
Xylanase	0.014^f^ ± 0.002
Protease	2.808^b^ ± 0,.002

^1^Values are expressed as mean ± standard deviation. Means followed by different superscript letters differ significantly according to Tukey’s test (p ≤ 0.05)

The enzymes recovered from the SMIA-2 strain were not purified before spray-drying, so the crude pectinase extract containing fermentation by-products (other proteins, carbohydrates, and salts) was also spray-dried to develop stable, dry formulations of the enzyme set. This fact could represent a significant economic saving in unit operations when the process is scaled up to industrial margins. Spray drying is a widely used, economical drying method for stabilizing enzymes (Assadpour and Jafari, 2019; Bajaj et al., 2021; Schutyser et al., 2012). However, when subjected to the high temperatures used in spray drying, some enzymes can be denatured and consequently lose their catalytic activity (Hamin et al., 2014). Various excipients are often used as bulking agents and protective additives to stabilize enzyme complexes (Emami et al., 2018; Ohtake et al., 2011). The optimal temperature for the pectinase activity produced by *Bacillus licheniformis* SMIA-2 was 70 °C. Thus, to minimize thermal denaturation that leads to the loss of pectinase activities, maltodextrin and microcrystalline cellulose–selected based on their function and safety–were incorporated into the enzyme complex before spray drying, and the interactive effect between their concentrations and the inlet air temperature (110 °C) was used to obtain maximum retention of enzyme activities. Adding 0.5–1.0% (w/v) of maltodextrin and microcrystalline cellulose before spray drying at an inlet air temperature of 110 °C resulted in a powder with good levels of pectinase activity, as observed when only cellulose was used as adjuvant (Cruz, 2021). Because the culture supernatant typically contains low solids, the use of excipients is justified to develop more stable enzyme formulations and improve dry mass recovery (Libardi et al., 2020).

Siqueira et al. (2013) selected the appropriate adjuvant and evaluated the best parameters for drying the enzymatic extract of *Trichoderma harzianum* containing peptidases and cellulases. Better enzyme activity was observed when the additives were incorporated before spray-drying the formulations, and the degree of improvement depended on the carbohydrate type and concentration. Using spray drying with additives, especially maltodextrin, was an efficient method for drying slaughterhouse rumen fluid to produce an environmentally friendly cellulase additive for animal feed (Sarteshnizi et al., 2018). The concentrated cellulase extract from *Trichoderma harzianum* TRIC03-LPBII was dried in a spray dryer with 20% (w/v) maltodextrin, yielding an enzymatic powder formulation with 85% stability after 60 days (Libardi et al., 2020).

According to the literature, numerous enzymes have already been dried in a spray dryer, such as α-amylases (Obón et al., 2020), lipases (Torres et al., 2016), peptidases (Cabral et al., 2017), cellulases (Libardi et al., 2020), β-galactosidases (Lipiäinen et al., 2018), xylanases (Gupta et al., 2014), phytases (Sato et al., 2014), and proteases (Bolzan et al., 2022). However, no studies have examined microbial pectinases subjected to spray drying, a focus of this study.

### Contextual comparison with reported pectinases and benchmark limitations

The application of enzymes in biotechnological processes depends on their products being highly active and stable for long periods, even when stored under non-ideal conditions (Namaldi et al., 2006; Samborska et al., 2005). Dehydrated enzyme formulations have advantages over aqueous crude enzymatic extracts, providing increased shelf life and practical handling (Costa-Silva et al., 2014). The extract rich in pectinases dried in the presence of 0.5% (w/v) of maltodextrin, 1.0% (w/v) of microcrystalline cellulose, and at an inlet temperature of 110ºC was more stable during storage at 5 °C than 25 °C, demonstrating that storing enzymes in powder form and at low temperatures prolongs their stability and shelf life, which is extremely important for use in industry.

Compared with previously reported studies, our crude-extract enzyme exhibited competitive performance, particularly in activity under alkaline conditions and at high temperatures. The optimal activity at pH 8.5 and 70 °C, along with the retention of 80% activity after prolonged thermal exposure, indicates stability superior to or comparable to that of many reported *Bacillus*-derived pectinases, which often exhibit lower thermal resistance or narrower pH ranges (Amin et al., 2019; Sadh et al., 2018). Our crude pectinase demonstrated activity under alkaline and moderately high-temperature conditions, which may represent an advantage for industrial applications requiring robust catalytic performance, since commercial fungal pectinases exhibit optimal activity under acidic conditions (pH 4.5–5.5).

The selected spray-drying combination not only preserved enzyme activity during cold storage for up to 180 days but also underscored the viability of eco-friendly production processes. However, the preservation of enzymatic structure following atomization cannot be conclusively confirmed. The assessment of spray-drying performance was based primarily on residual pectinase activity and post-drying storage stability. Although the retained enzymatic activity suggests that the drying conditions were sufficiently mild to preserve catalytic function to a considerable extent, the possibility of partial structural modification or protein denaturation cannot be excluded. Future studies involving electrophoretic and proteomic analyses are proposed to further evaluate enzyme integrity after spray drying.

The study aligns with the Sustainable Development Goals (SDGs), particularly SDG 9 (Industry, Innovation and Infrastructure), by promoting technological advancement; SDG 12 (Responsible Consumption and Production), by reducing waste and encouraging resource efficiency; and, indirectly, SDG 13 (Climate Action), through the implementation of low-impact, sustainable practices. Further experiments on proteome sequencing are needed to discuss the final biotechnological applications of the techniques described herein.

Genomic analysis and in silico annotation performed in this study do not constitute direct experimental validation of pectinase gene expression, secretion, or enzymatic function. The identification of putative pectinase-related genes, operon organization, and secretion signals was based on bioinformatic predictions and comparative genomic analyses. Although extracellular pectinolytic activity was experimentally detected during fermentation, complementary approaches such as RT-qPCR, proteomic analysis, secretome profiling, zymography, or purification and biochemical characterization of the enzyme were not performed in the present study. These additional validation strategies are recognized as important future steps for confirming the functional role of the predicted pectinase-associated genes.

The present work shows that *Bacillus licheniformis* SMIA-2 produced a pectinase-active crude enzyme preparation using passion fruit peel flour and corn steep liquor, which could be converted into a refrigerated, spray-dried powder with retained pectin-directed activity. Reanalysis of the draft genome identified five candidate pectinase-related loci and a plausible uronate-catabolic pathway, providing hypotheses to explain the observed phenotype. However, because genome-based predictions were not experimentally linked to individual enzymes and no purified-enzyme or commercial-benchmark comparisons were performed, the data support the potential of SMIA-2 as a sustainable pectinase-production platform rather than making definitive claims about gene function or industrial superiority.

## Supplementary Information

Below is the link to the electronic supplementary material.Supplementary file1 (DOCX 83 KB)

## Data Availability

The datasets generated during and/or analyzed during the current study are available from the corresponding author on reasonable request. The genome sequence analyzed in this work is publicly available under GenBank accession JAACZZ000000000.
