# Supplementary material for: Scalable production of pectinases from Bacillus licheniformis SMIA-2 using agro-Industrial by-products with genomic insights
Source: Food Sci Biotechnol. 2026 Jul 25;35(11):3349–59. doi: 10.1007/s10068-026-02252-3 (PMC13530104; doi:10.1007/s10068-026-02252-3)
Supplement: Supplementary file 1 [file 10068_2026_2252_MOESM1_ESM.docx]

***Scalable Production of Pectinases from* Bacillus licheniformis *SMIA-2 Using Agro-Industrial By-Products with Genomic Insights***

Erica CRUZ^1,2^, Larissa Pacheco FERREIRA^3^, Albert Remus Romero ROSANA^4^, Samara Pinto Custódio BERNARDO^5^, Meire Lelis Leal MARTINS^6^, João Paulo FABI^7,8*^

^1^Department of Food Science and Experimental Nutrition, School of Pharmaceutical Sciences, University of São Paulo, Brazil; <https://orcid.org/0000-0002-5264-6116>

^2^Food Technology Laboratory, State University of North Fluminense, Campos dos Goytacazes, Rio de Janeiro, Brazil. <https://orcid.org/0000-0002-5264-6116>

^3^Food Technology Laboratory, State University of North Fluminense, Campos dos Goytacazes, Rio de Janeiro, Brazil. <https://orcid.org/0000-0001-6231-8282>

^4^Department of Biology, De La Salle University, Taft Avenue, Manila, The Philippines. <https://orcid.org/0000-0001-7847-7763>

^5^Food Technology Laboratory, State University of North Fluminense, Campos dos Goytacazes, Rio de Janeiro, Brazil. https://orcid.org/0000-0003-2579-1307

^6^Food Technology Laboratory, State University of North Fluminense, Campos dos Goytacazes, Rio de Janeiro, Brazil. <https://orcid.org/0000-0002-9812-0232>

^7^Food and Nutrition Research Center (NAPAN), University of São Paulo, São Paulo, Brazil. <https://orcid.org/0000-0002-7430-7756>

^8^Food Research Center (FoRC), CEPID-FAPESP (Research, Innovation and Dissemination Centers, São Paulo Research Foundation), São Paulo, São Paulo, Brazil. <https://orcid.org/0000-0002-7430-7756>

***Corresponding author:**

João Paulo Fabi

Email: jpfabi@usp.br

**Supplementary Figure 1A.** Standard curve for galacturonic acid using the DNS protocol.

**Supplementary Figure 1B.** Standard curve for glucose using the DNS protocol.

**Supplementary Table 1.** Effect of storage time on the activity of pectinase powders at 5ºC to 6 months.

| **Time Days** | **U/mL** |
| --- | --- |
| 0 | 13.659 ^a^ ± 0.003 |
| 30 | 13.581 ^b^ ± 0.002 |
| 60 | 13.569 ^c^ ± 0.001 |
| 90 | 13.560 ^d^ ± 0.003 |
| 120 | 13.554 ^e^ ± 0.003 |
| 150 | 13.549 ^f^ ± 0.001 |
| 180 | 13.536 ^g^ ± 0.001 |

^1^Means with the same letters in the same column do not differ significantly at p ≤ 0.05, according to the Tukey test.
